# Unraveling the diversity of hyphal explorative traits among *Rhizophagus irregularis* genotypes

**DOI:** 10.1007/s00572-024-01154-8

**Published:** 2024-06-03

**Authors:** Daquan Sun, Martin Rozmoš, Vasilis Kokkoris, Michala Kotianová, Hana Hršelová, Petra Bukovská, Maede Faghihinia, Jan Jansa

**Affiliations:** 1 Institute of Microbiology, Czech Academy of Sciences, Vídeňská, 14220, Praha 4, 1083 Czech Republic; 2 Vrije Universiteit Amsterdam, Amsterdam Institute for Life and Environment (A-LIFE), De Boelelaan 1108, Amsterdam, NL-1081HZ The Netherlands; 3 Department of Plant Pathology, Entomology, and Microbiology, Iowa State University, 2213 Pammel Dr, Ames, IA 50011 US

**Keywords:** Hyphosphere microbiome, Intraspecific differences, Mycorrhizal hyphal networks, Quantitative real-time PCR (qPCR), Soil nitrogen exploration, Stable isotopic labeling and tracing

## Abstract

Differences in functioning among various genotypes of arbuscular mycorrhizal (AM) fungi can determine their fitness under specific environmental conditions, although knowledge of the underlying mechanisms still is very fragmented. Here we compared seven homokaryotic isolates (genotypes) of *Rhizophagus irregularis*, aiming to characterize the range of intraspecific variability with respect to hyphal exploration of organic nitrogen (N) resources, and N supply to plants. To this end we established two experiments (one in vitro and one in open pots) and used ^15^N-chitin as the isotopically labeled organic N source. In Experiment 1 (in vitro), mycelium of all AM fungal genotypes transferred a higher amount of ^15^N to the plants than the passive transfer of ^15^N measured in the non-mycorrhizal (NM) controls. Noticeably, certain genotypes (e.g., LPA9) showed higher extraradical mycelium biomass production but not necessarily greater ^15^N acquisition than the others. Experiment 2 (in pots) highlighted that some of the AM fungal genotypes (e.g., MA2, STSI) exhibited higher rates of targeted hyphal exploration of chitin-enriched zones, indicative of distinct N exploration patterns from the other genotypes. Importantly, there was a high congruence of hyphal exploration patterns between the two experiments (isolate STSI always showing highest efficiency of hyphal exploration and isolate L23/1 being consistently the lowest), despite very different (micro) environmental conditions in the two experiments. This study suggests possible strategies that AM fungal genotypes employ for efficient N acquisition, and how to measure them. Implications of such traits for local mycorrhizal community assembly still need to be understood.

**Supplementary Information:**

The online version contains supplementary material available at 10.1007/s00572-024-01154-8.

## Introduction

Arbuscular mycorrhizal (AM) fungi establish symbiotic partnerships with a vast number of terrestrial vascular plants, playing a role in upholding biodiversity and facilitating essential resource exchanges within ecosystems (Johnson et al. 2012; Lekberg et al. 2024; Martin and van der Heijden 2024). In this often-mutualistic relationship, plants provide AM fungi with reduced carbon generated through photosynthesis while AM fungi reciprocate with crucial nutrients such as phosphorus (P) and nitrogen (N) from the soil. Despite the multitude of plant species involved in this symbiotic partnership, the diversity of AM fungal species is notably lower, and multiple AM fungal species have been observed to coexist and colonize the same plant simultaneously (Brundrett 2009; Rodríguez-Echeverría et al. 2017; Mathieu et al. 2018). This intricate interaction between AM fungi and host plants presents a range of dynamic scenarios including shifts from positive to negative outcomes of the symbiosis under stressful environmental conditions (Hahn et al. 2018; Sendek et al. 2019; Kokkoris and Hart 2019), host influence on AM fungal genetics (Kokkoris et al. 2021; Cornell et al. 2022), differences in AM fungal community composition associated with different host plants (Castelli and Casper 2003; Koch et al. 2017), and influence of host plant genetic diversity and root morphology on AM fungal colonization rates (Wen et al. 2019; Aavik et al. 2021). These complexities underscore the significance of understanding AM fungal-plant interactions in nutrient and carbon exchanges within ecosystems (Lekberg et al. 2024).

When exploiting organic nutrient (P or N) sources, AM fungi rely on the assistance of other soil microbes such as bacteria and protists for mineralization of those complex nutrient resources (Zhang et al. 2016; Bukovská et al. 2018; Rozmoš et al. 2022). The microbiome close to or on AM hyphae (the so called hyphosphere microbiome) possibly could be specific to certain or to all AM fungi (Emmett et al. 2021; Sun et al. 2023; Faghihinia et al. 2024) and this association between AM fungi and their hyphosphere microbiota is likely the key to overcome the lacking exoenzyme repertoire of the AM fungi that prevents them from alone accessing such resources (Tisserant et al. 2013).

Diversity of AM fungal communities can be aligned with their ecological functions (Sanders and Rodriguez 2016). Functional diversity among AM fungal species with respect to efficiency of nutrient acquisition and the carbon costs borne by the host plant has been long recognized (Cavagnaro et al. 2005; Koch et al. 2006; Jansa et al. 2008; Lendenmann et al. 2011; Giovannini et al. 2020). Besides, there obviously are major differences in functional traits among isolates of the same AM fungal species (Munkvold et al. 2004), most likely because of genetic and/or epigenetic differences (Kiers et al. 2011; Mathieu et al. 2018; Peña et al. 2020). Some argue that the effects on plants of the intraspecific diversity of AM fungi may equal or even surpass those of interspecific diversity (Johnson et al. 2012). Recent findings have explained that some of the genomes of such strains are phylogenetically related but differ in structure, gene content and epigenetic modifications (Sperschneider et al. 2023). In line with this, substantial genotypic and functional differences have been found among isolates of the same species (Börstler et al. 2008; Avio et al. 2009; Novais et al. 2014; Aavik et al. 2021). The extensive intraspecific diversity observed in characteristics such as mycelium growth patterns and the enhancement of P uptake by the host plant is believed to serve as a compensatory mechanism in AM community function, particularly in cases where AM fungal species diversity is low (Munkvold et al. 2004). Alternatively, the intraspecific diversity of AM fungi itself can exert a significant influence on host plants (Croll et al. 2008; Angelard et al. 2010; Novais et al. 2014). Moreover, recombination events between certain AM genotypes, especially in constrained environments, have been identified, contributing to the already high levels of intraspecific variation within AM fungi (Croll et al. 2009; Mathieu et al. 2018; Sperschneider et al. 2023). It is important to note that differing habitats or soil properties also may induce changes in AM populations, even if they do not directly correlate with AM intraspecific phenotype expressions (Ehinger et al. 2009).

While intraspecific diversity within AM fungi often has been linked to factors such as soil P acquisition (Novais et al. 2014) and adaptation to changing environmental conditions (Behm and Kiers 2014), there remains a knowledge gap of how intraspecific diversity specifically influences broad nutrient acquisition processes. The morphology of AM fungal mycelium reacting to environmental changes mistakenly can be considered as intraspecific phenotypic plasticity. This potential misunderstanding may hinder our exploration of whether nutrient acquisition is modulated genetically at the intraspecific level, possibly from host plant-mediated processes or unique strategies developed by distinct AM fungal genotypes.

**Fig. 1:**
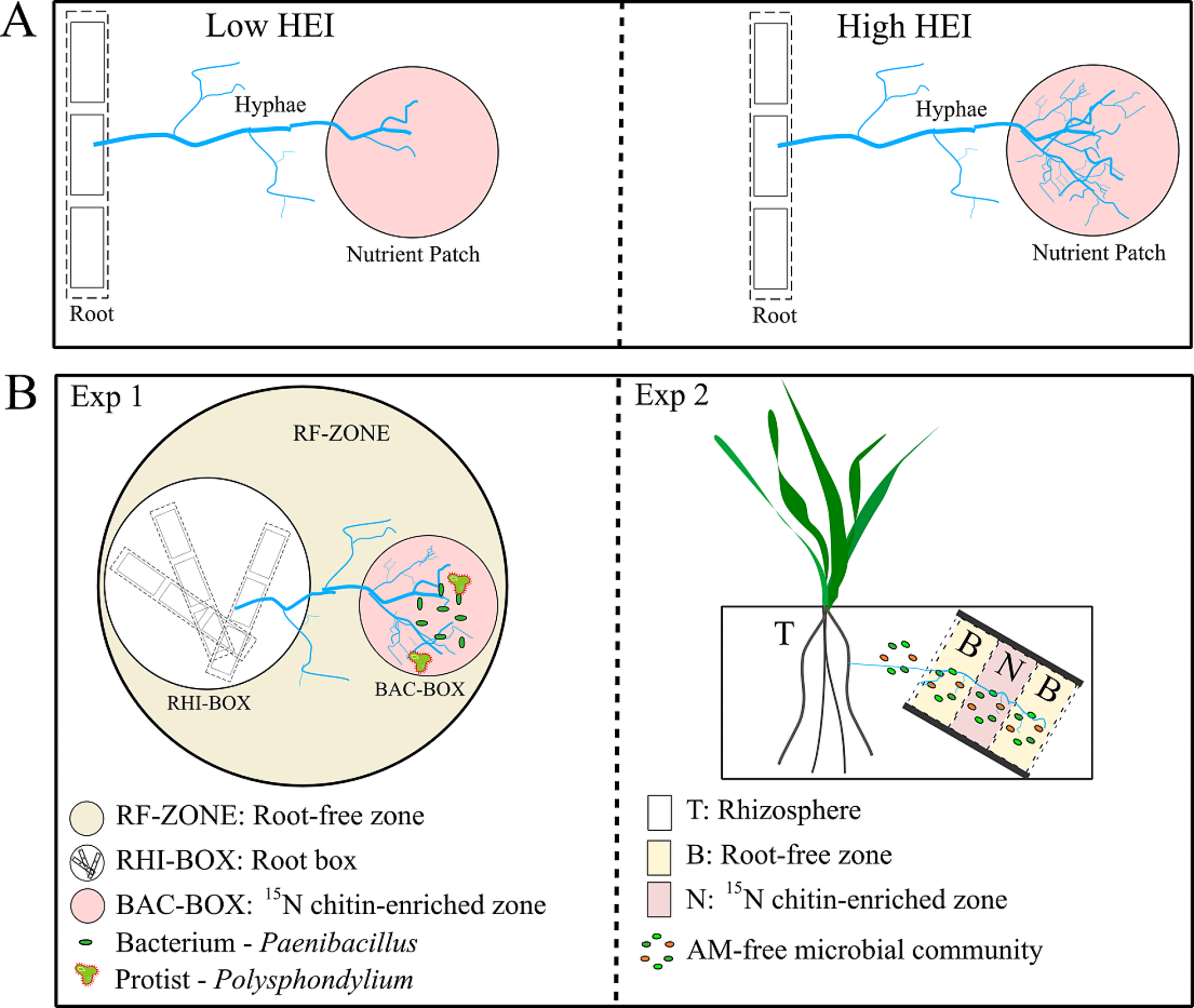
Possible strategies of soil nutrient exploration by arbuscular mycorrhizal (AM) fungi, with respect to development of their extraradical hyphae in a nutrient enriched zone (shown in pink) outsides of roots (**A**). These could be described by a hyphal exploration index (HEI), where a low value corresponds to nutrient-unresponsive hyphal networks and a high value corresponds to genotypes/species/genera responding to the nutrients by intensive hyphal exploration/branching within the zone. The differences in HEI across genotypes of AM fungus *Rhizophagus irregularis* were evaluated in two experimental set-ups (**B**), one in vitro (Exp 1) with a root zone (RHI-BOX), delimited from the rest by a 40 μm nylon mesh, a root-free (RF) zone, and a zone (BAC-BOX) enriched with organic nitrogen (^15^N-labeled chitin) and added with a chitinolytic *Paenibacillus* sp. with or without a protist (*Polysphondylium* sp.). A follow up experiment (Exp 2) was carried out in pots and contained a nutrient enriched zone with ^15^N-labeled chitin (N, hyphosphere compartment) delimited from the rhizosphere (T) by a buffer zone (B) and a 40 μm mesh (similar to Faghihinia et al. 2024)

To improve our understanding of intraspecific functional diversity in arbuscular mycorrhizas in terms of nutrient acquisition, we compared the strategy of seven different genotypes of *Rhizophagus irregularis* in exploring organic N-enriched root-free patches and in transferring the N from such patches to their plant host (as depicted in Fig. 1A). *R. irregularis* strains differ dramatically in gene content and epigenetics (including methylation and chromosome conformation) (Yildirir et al. 2022), which might be the primary source of variation in downstream effects on N utilization. The fungi had access to organic N either in a BAC-BOX of an in vitro experiment (Fig. 1B left panel) or a root-free zone in a pot experiment (Fig. 1B, right panel). In both experiments, we supplied microorganisms carrying out N mineralization in the organic N source zone, and measured plant growth and N nutrition as well as ^15^N transfer from the organic amendment to plants via the mycorrhizal pathway.

**Fig. 2:**
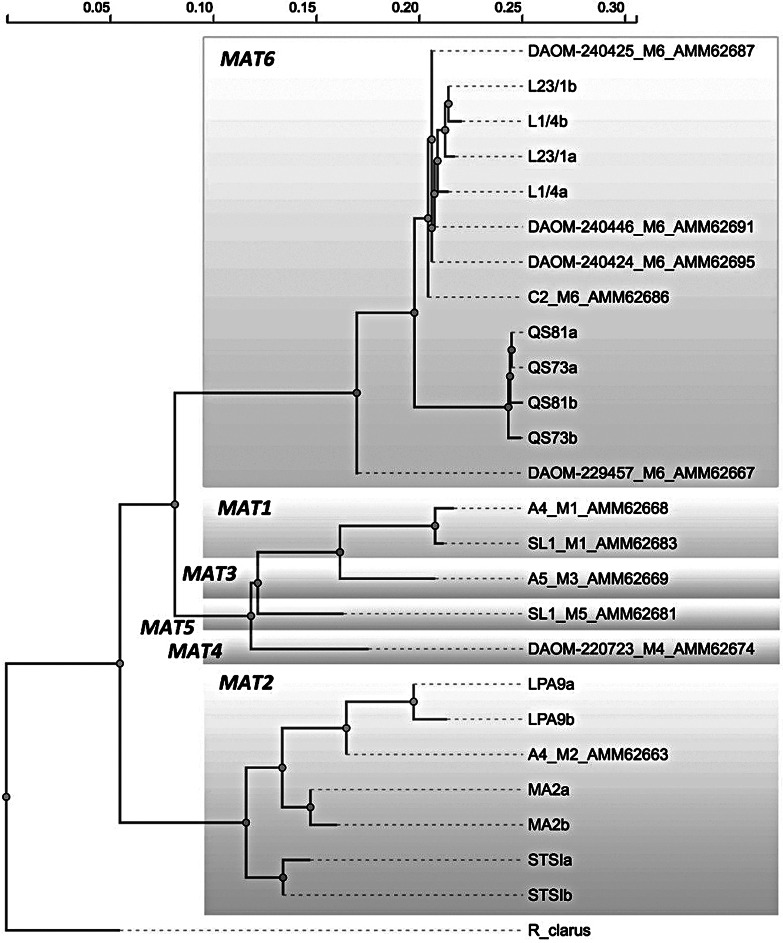
Phylogenetic tree constructed from aminoacid sequences of mating (MAT) genes homeodomain (HD) 2 obtained from the different *Rhizophagus irregularis* genotypes in this study (designated by their isolate code and “a” and “b” for the two replicate sequences per each fungal isolate) and corresponding reference sequences (designated by their accession numbers starting with “AMM62”) downloaded from GenBank. *Rhizophagus clarus* MUCL-46238 sequence (Genbank accession number AMM62664, labeled as “R_clarus”) was used as an outgroup to root the tree. Different MAT gene variants (MAT1 – MAT6) as described earlier (Ropars et al. 2016) are indicated. Both sequence alignment and phylogenetic tree construction were carried out using Clustal Omega (www.ebi.ac.uk/jdispatcher/msa/clustalo)

## Materials and methods

### Biological materials

In the research described here, seven *Rhizophagus irregularis* genotypes (hereafter referenced as L1/4, L23/1, LPA9, MA2, QS73, QS81, and STSI, more details in supplementary Table S1) were used, associated either with Ri T-DNA transformed chicory roots under in vitro conditions (Exp 1) or with *Andropogon gerardii* (a C4 AM host plant that is tolerant to environmental stresses such as drought) plants in pots (Exp 2). Genotyping of mating type (MAT) genes of the different isolates and their subsequent sequencing was carried out as described previously (Sperschneider et al. 2023). Gene sequences were deposited in GenBank under accession numbers PP721335-PP721348. The (non-mycorrhizal, NM) chicory root culture originally was provided by S. Declerck (https://www.mycorrhiza.be/ginco-bel) and maintained on solidified MSR medium as reported previously (Rozmoš et al. 2022). Seeds of *Andropogon* were purchased from Jelitto Staudensamen GmbH (https://www.jelitto.com). For the in vitro Exp 1, a culture of *Paenibacillus chitinolyticus* CCM 4527 was purchased from the Czech microbial culture collection (https://ccm.sci.muni.cz), maintained on solid lysogeny broth (1.5% agar) and stored in 30% glycerol at -80 °C prior to use. The protist *Polysphondylium pallidum* was maintained on water agar using an *Escherichia coli* cell suspension spread on the surface, with the bacteria being produced previously on lysogeny broth agar, as reported earlier (Rozmoš et al. 2022).

### ^15^N-Labeled chitin production

Isotopically (^15^N) labeled chitin was obtained from cell walls of a pure culture of *Zygorhynchus* sp. grown in liquid mineral media with the (NH_4_)_2_SO_4_ being the sole N source for the saprotrophic fungus, as described previously (Bukovská et al. 2018). Briefly, the chitin batch for Exp 1 contained 5.5% N and 45.3% C by weight. The chitin batch for Exp 2 contained 5.3% N and 45.8% C by weight. The N in both chitin batches was fully isotopically labeled (> 98 atom% represented by ^15^N).

### Experiment 1 (in vitro)

This experiment was carried out to compare the seven *R. irregularis* genotypes in terms of their hyphal growth into the root-free zone, their capabilities in exploring the ^15^N-labeled chitin resource placed in the root-free compartment (BAC-BOX), their utilization of the N source in concert with other microorganisms (bacteria), and the effect of protistan amendment into the BAC-BOX (Fig. 1B, left panel). This required the following set-up: The experiment was carried out in the in vitro cultivation vessels (microcosms), each consisting of a large, sterile polystyrene Petri dish (diameter 15 cm, 2 cm height) and two smaller compartments embedded into the microcosm, as described previously (Rozmoš et al. 2022). One of the small compartments was the RHI-BOX, provided with chicory roots pre-colonized or not with a specific AM fungal genotype at the beginning of the experiment (Fig. 1B, left panel). The RHI-BOX was made from a lid of a small (6 cm diameter) polystyrene Petri dish with a hole for root transfer drilled into its top, and the bottom opening sealed with a 42 μm nylon mesh (Silk & Progress, Brněnec, Czech Republic). The rim of the box was dipped in chloroform before pressing it against the mesh, which firmly glued the box walls to the mesh. After preparing the RHI-BOXes, they were sterilised by γ-rays (> 25 kGy, Bioster, Veverská Bítýška, Czech Republic). The second smaller compartment (hereafter termed the BAC-BOX) was made from the bottom of a small (6 cm diameter) Petri dish and placed at a distance of at least 5 mm from the RHI-BOX. The large Petri dish was filled with 100 ml of standard modified Strullu and Romand (MSR) medium, pH 5.5 (Cranenbrouck et al. 2005), containing 93 µg P and 379 µmol N, plus 1% (w: v) sucrose, and solidified with 0.3% (w: v) gelling agent (Phytagel, Merck, Darmstadt, Germany). The P and N concentrations in Phytagel powder were 560 µg g ^− 1^ and 46 µmol g ^− 1^, respectively. The sterile labeling compartment (BAC-BOX) was inserted into the liquid medium freshly poured into the large Petri dish before it solidified and was held down by a sterile metal plug. The rim of the BAC-BOX protruded at least 1 mm above the medium (Rozmoš et al. 2022). After the medium solidified, the RHI-BOX was placed on top of the MSR medium, and the metal plug was removed from the BAC-BOX. Mycorrhizal or NM chicory roots were added to the RHI-BOX through the hole and incubated at 24 °C in darkness for 64 days (16 replicate microcosms established per each fungal treatment). After this period, the roots filled the RHI-BOX and AM fungal hyphae colonized the entire volume of the MSR medium. Thereafter, the BAC-BOX was filled with 15 mg of the ^15^N-chitin (containing 55 µmol N and 135 µg P) and 5 mL of N-free MSR medium without sucrose and containing 0.3% phytagel similarly as in Rozmoš et al. (2022). After 21 days of additional incubation, 60 µL of actively growing *Paenibacillus* suspension (OD600 = 0.5) was added to the center of the BAC-BOX. After another 16 days, a suspension of *Polysphondylium* spores was added or not to the bacterial colony (8 replicates of each fungal treatment added with the protist suspension and 8 replicates just with sterile water) and the microcosms were incubated for another 17 days. The experiment thus was designed as two-factorial with fully crossed treatments of each of seven *R. irregularis* genotypes and a NM control versus *Polysphondylium* amendment (+ or -) = 16 treatments, with 8 replicates per treatment.

### Experiment 2 (pot)

The pot experiment was set up to verify whether the differences observed among the AM fungal genotypes in terms of their nutrient exploration patterns under in-vitro conditions also held under open pot conditions with complex microbiomes present. Like Exp 1, the pot experiment contained three zones, namely the rhizosphere, the buffer zone, and the hyphosphere (Fig. 1B, right panel). The hyphosphere was enriched with ^15^N-chitin. The experiment thus contained only one experimental factor, namely no inoculation or inoculation with a specific AM fungal genotype (NM, or one of the seven *Rhizophagus* genotypes, respectively), replicated four times per treatment, and the pots arranged in a completely randomized design in a glasshouse.

The experiment was carried out in 2 L tall pots (volume = 20 cm × 11 cm × 11 cm), each filled with 2.3 kg (dry weight) of sterilized soil-sand-zeolite mixture (10:45:45, v:v:v), which has been thoroughly described previously (Bukovská et al. 2016; Gryndler et al. 2018). Briefly, the potting substrate was coarsely structured, slightly alkaline (pH = 8.9 in a water slurry 1:2.5, w: v) and nutrient-poor. It contained 46.5 mg kg ^− 1^ total P, of which 2.6 mg kg ^− 1^ was water-extractable (1:10 w:v, shaken for 20 h, and filtered through 0.2 μm nitrocellulose mixed ester filter), as well as 0.013% and 0.22% total N and organic C, respectively (Jansa et al. 2020).

All substrate per pot (including that added to the root-free compartments, see below) was supplemented with indigenous soil microbes in the form of 20 mL pot^− 1^ of sieved NM inoculum slurry (i.e., potting mixture from open pots planted to leeks and devoid of AM fungi, aged 3.5 years, mixed with sterile water 1:10, v:v, and sieved through 500 μm steel mesh to remove plant roots and coarse debris), and 10 mL pot ^− 1^ of 5 μm filtrate from mycorrhizal potting substrate. The latter was obtained from pots containing *R. irregularis* BEG 236 planted to leeks, aged 3 years, mixed with sterile water 1:10 (v:v) and filtered through Teflon filters with 5 μm pores (Gryndler et al. 2018; Jansa et al. 2020; Bukovská et al. 2021). All the volume of the pot was accessible to the roots except a small root-free compartment made of PVC tubing (3.6 cm in diameter, 3 cm length), containing 40 g of the potting substrate separated from the rooted pot volume with a 40 μm nylon mesh on both ends. In the middle of the root-free compartment, a 4 g section of the substrate, amended with ^15^N-chitin (Bukovská et al. 2018) was placed, delimited by two discs of a coarse nylon mesh, 200 μm mesh size (Fig. 1B, right panel). The root-free compartment was inserted in each pot approximately 8 cm below the substrate surface, at about 5 degrees of slant (Fig. 1B, right panel) to prevent water accumulation in the compartments and to facilitate ingrowth of AM fungal hyphae. Twenty mg of self-made, fully ^15^N-labeled chitin were added to each root-free compartment, representing the inputs of 84 µmol N and 177 µg P per ingrowth compartment. After adding the chitin powder to the root-free compartment (hyphosphere), 400 µl sterile deionized water was poured over it to allow its incorporation into the 4 g middle section (Faghihinia et al. 2024). Pots were each seeded with 50 seeds of *A. gerardii* and kept in the glasshouse for 45 days under conditions detailed elsewhere (Faghihinia et al. 2024). Briefly, plants were maintained under natural lighting supplemented with 500 W metal halide lamps, with the glasshouse temperature ranging between 18 °C at night and 37 °C during the warmest days. Pots were watered with deionized water to maintain gravimetric water content between 18% and 24% (corresponding to 60% and 80% of the water holding capacity of the substrate). No additional fertilizers were provided throughout the cultivation.

### Harvest and analyses

#### Experiment 1

Upon harvest, the chicory roots from the RHI-BOX were collected, dried at 65 °C for 3 days, weighed and then pulverized in a ball mill (MM 200, Retsch, Haan, Germany). Thereafter, C and N concentrations and isotopic composition of N were measured using a Flash 2000 elemental analyzer coupled with a Delta V Advantage isotope-ratio mass spectrometer (Thermo Fischer Scientific, Bremen, Germany), using 2 (± 0.3) mg aliquots, weighed on a 6-digit balance and wrapped in tin capsules. The hyphae from the root-free zone were collected, after dissolution of the phytagel in a citrate buffer, on an Omnipore membrane filter (5 μm pore size, 47 mm diameter, Merck Millipore, Burlington, MA, USA). Dry weights of the hyphae were recorded after drying them in a vacuum centrifuge for 2 days, and their C and N concentrations and N isotopic compositions were analyzed similarly as for the roots. DNA was extracted from the lyophilized contents of BAC-BOXes as described previously (Rozmoš et al. 2022), using homogenization of the samples in ceramic mortars, spiking the samples with internal DNA standard (Thonar et al. 2012), and extracting the DNA with the glass-milk method (Gryndler et al. 2014).

#### Experiment 2

Shoots and roots were harvested separately from each pot. Shoots were dried at 65 °C for 3 days and weighed. The roots were washed from the rhizosphere compartment with tap water, briefly rinsed with deionized water, blotted against a paper towel, and weighed fresh. Thereafter, a representative aliquot of the fresh roots from each pot was transferred to 50% ethanol for staining in Trypan Blue (Koske and Gemma 1989) and the colonization was assessed microscopically according to McGonigle et al. (1990). All the remaining roots were weighted fresh and then dried to constant weight at 65 °C for 3 days and weighed. The dried plant samples were subsequently pulverized in a MM200 ball mill at 25 Hz for 2 min before subsequent elemental and molecular analyses.

Samples of the substrates were collected from the compartments including the rhizosphere (T), the buffering zone (B), and the hyphosphere (N). Substrates were dried at 65 °C for 3 days and milled using the ball mill as above, before further elemental and molecular analyses.

Root (~ 10 mg dry powder) and substrate (~ 600 mg dry powder) samples were used to extract DNA using the Plant DNeasy kit (Qiagen, Venlo, Netherlands) and DNeasy PowerSoil kit (Qiagen), respectively. To determine DNA extraction efficiency, an internal DNA standard spike containing 2 × 10^10^ gene copies of cassava mosaic virus gene fragment (Thonar et al. 2012) was added to each sample prior to DNA extraction.

Total P in plant shoot and root (0.1 g each) were determined by the malachite green method (Ohno and Zibilske 1991) after dry incineration of the samples (550 °C, 12 h) and extraction of the ashes with concentrated nitric acid (Püschel et al. 2017).

The C and N concentrations in the plant biomass (2 mg) and substrates (20 mg), as well as the ^15^N enrichment in the different samples were analyzed using the Flash EA 2000 elemental analyzer coupled to the isotope ratio mass spectrometer as above.

### Quantitative real-time PCR

Quantitative real-time PCR (qPCR) was used to analyze the development of the AM fungi in both experiments, as well as for the quantification of bacteria and protists in Exp 1. To this end, we carried out four independent (single-plex) qPCR assays using the DNA extracted from BAC-BOXes of Exp 1, employing the primers/TaqMan probes “ISC”, “mt5”, “Eub”, and the “Polysphondylium F2/R2” to quantify the recovery of the internal DNA standard, the development of *R. irregularis* by targeting its mitochondrial large ribosomal subunit (mtLSU), bacterial 16 S, and protistan 18 S genes, respectively. The different assays including their calibration were described in detail previously by Dudáš et al. (2022) and Rozmoš et al. (2022). The qPCR in the root and substrate samples from Exp 2 aimed at quantification of the AM fungi, using primers/TaqMan probes “intra” and “mt5”, targeting nuclear and mitochondrial LSU genes of *Rhizophagus*, respectively (Thonar et al. 2012; Couillerot et al. 2013) besides the ISC primers + TaqMan probe to measure internal DNA standard recovery. Additionally, based on *de novo* Sanger sequencing of the mtLSU from various *Rhizophagus* isolates (sequences deposited in NCBI under accession numbers OR947632-OR947644) we designed new primers with a TaqMan probe to specifically detect QS81 and similar isolates such as QS73 (forward: TACCTATGCCGCTACG; reverse: GCTTCCACAATATTATATCATG, TaqMan labeled with FAM and BHQ2 quencher: TTTTCAACCATGTTTAGACCA). This marker was then used to measure abundance of the QS81 sequence motif in the different root and substrate samples in Exp 2, calibrating the assay with PCR amplicon generated from the QS81 isolate as described previously (Thonar et al. 2012). The qPCR quantification was carried out in 96-well plates using a 20-µL final reaction volume. Either Luna universal probe qPCR master mix (M3004; for assays including a TaqMan probe) or Luna universal qPCR master mix (M3003; without a probe) provided by New England Biolabs (Ipswich, MA, USA) were used. Fluorescence was recorded in the SYBR green/fluorescein color channel. By calculating the loss of the internal DNA standard upon DNA extraction, qPCR assays were normalized using the ISC recovery measured for each sample (Thonar et al. 2012).

### NGS sequencing

Only microbial communities from the substrate samples collected in rhizosphere (T) and hyphosphere (N) zones of Exp 2 were included in a high-throughput sequencing analysis. Details of amplicon library preparation for bacteria and archaea (primers 515–806) and for protists (primers V4) were described previously (Dudáš et al. 2022) and that of fungi (employing a semi-nested procedure with ITSOF-ITS4 and gITS7-ITS4 primers) recently by Faghihinia et al. (2024). The library pool was purified by paramagnetic beads and sequenced on the Illumina 2 × 300 platform at the Joint Microbiome Facility (JMF, Vienna, Austria).

Raw sequences were demultiplexed and adapter-trimmed, primers removed, quality filtered and clustered at 97% similarity in the Seed 2.0 software (Větrovský et al. 2018) as described previously (Dudáš et al. 2022). Most abundant sequences from each cluster were then identified by using the SILVA (prokaryotic and fungal) or PR2 (protistan) sequence databases to identify contaminants. Contaminants (such as chloroplast and mitochondria sequences within the prokaryotic dataset) were removed, samples rarefied to equal sequencing depth, sequences clustered again at the 97% similarity level, and the most abundant sequences per cluster re-identified. Relative abundances of the different microbial taxa (clumped at genus level instead of 97% similarity levels) per sample were then used for subsequent statistical analyses. The sequences were deposited in the Sequence Read Archive of the NCBI under the accession number PRJNA977454.

### Calculations and statistical analyses

Nutrient concentrations were used to calculate nutrient contents per plant/microcosm, using data of dry plant biomass per microcosm. Further, we calculated a hyphal exploration index (HEI) to describe whether a particular AM fungus proliferated in the nutrient-enriched patch or not as follows:

For experiment 1:

$$\text{HEI} =\frac{{\text{mtLSU\,gene\,copy\,numbers\,in\,the\,BAC {\text{-}} BOX}}}{{\text{mycelium\,dry\,biomass\,in\,the\,root {\text{-}} free\,(RF)\,zone}}}$$

For experiment 2:

$$\text{HEI} =\frac{{\text{mtLSU\,gene\,copies\,per\,unit\,weight\,of\,the\,hyphosphere\,(N)}}}{{\text{mtLSU\,gene\,copies\,per\,unit\,weight\,of\,the\,buffer\,(B)\,zone}}}$$

HEI for the NM control treatments was not calculated.

Conformity with the assumptions of analysis of variance (ANOVA) were checked for the different data by Shapiro-Wilk test for normality, Levene’s test for homogeneity of variances, or autocorrelation plots for possible interdependencies, all using the “car” package in R 4.2.2. (R Core Team 2022). If satisfying ANOVA assumptions, data were analyzed using either one- or two-way ANOVA (the latter only for the Exp 1), followed by post hoc Tukey’s honestly significant difference (HSD) tests if *p* (ANOVA) < 0.05. If conditions of ANOVA were not met, non-parametric Kruskal-Wallis tests (*p* < 0.05) were applied, followed by Dunn tests to separate treatment medians, provided the Kruskal-Wallis test was significant.

For Exp 2, permutational multivariate analyses of variance (perMANOVA) tested the effects of compartment (hyphosphere vs. rhizosphere) and the AM fungal treatment (including the NM control treatment) and their interaction on the beta diversity (Adonis, Bray) of bacterial, fungal and protistan communities with the ‘vegan’ package in R. The number of permutations in these tests was set at 10,000. Nonmetric multidimensional scaling (NMDS) ordinations of Bray-Curtis dissimilarities of microbial communities in the compartments of hyphosphere and rhizosphere were conducted. In case the stress value was too close to zero, indicative of overfitting, weighted classical (metric) multidimensional scaling (WCMD) was used instead of NMDS.

## Results

### Experiment 1

**Fig. 3:**
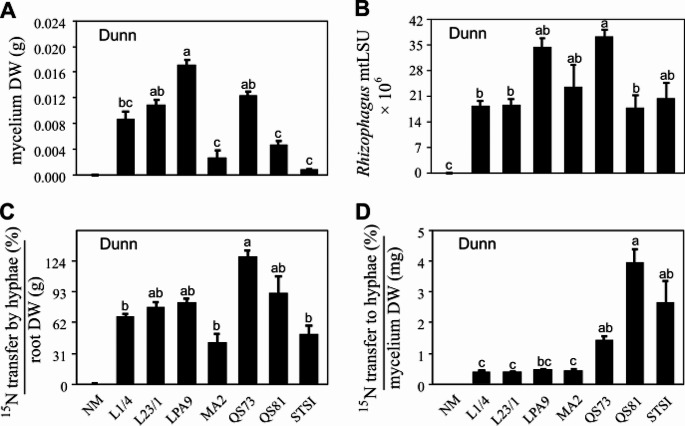
Arbuscular mycorrhizal (AM) fungal biomass, and ^15^N movement in **Exp 1** (**A**) Dry biomass (DW) of the mycorrhizal mycelium in the root-free zone per microcosm; (**B**) *Rhizophagus* (mitochondrial large ribosomal subunit gene, mtLSU, detected by mt5 marker, gene copies per BAC-BOX); (**C**) ^15^N taken up by the mycorrhizal hyphae from the BAC-BOX compartment and detected either in the roots or the hyphae collected from the root-free zone, per unit DW of roots, and (**D**) ^15^N detected in the hyphae collected from the root-free zone and standardized per unit DW of the hyphae. Treatments include non-mycorrhizal (NM) control and seven genotypes (isolates) of AM fungal species *Rhizophagus irregularis* (L1/4, L23/1, LPA9, MA2, QS73, QS81, and STSI). Bars represent means (12 to 14 biological replicates), error bars represent standard errors. Bars topped by the same letter do not differ significantly according to the non-parametric Kruskal-Wallis test (followed by a Dunn’s pairwise multiple comparisons procedure to separate treatment medians, *p* < 0.05). When no letter is assigned to the NM treatment, the NM control was excluded from the analysis (because of missing data or ratio values would include a zero denominator)

Measured parameters were mostly affected by the identity of the AM fungal genotype and less by the presence or absence of the protists, or the interaction between the two experimental factors (Table S2). The genotypes LPA9, QS73 and L23/1 had higher hyphal biomass than the other genotypes in the root-free zone (Fig. 3A). In the BAC-BOX, the highest abundance of *Rhizophagus* mtLSU was observed in the QS73 genotype, where it was significantly higher than in L1/4, L23/1, and QS81 genotypes (Fig. 3B). Significant effects of protist inoculation were observed on bacterial and protistan abundances (Table S2). The amount of ^15^N transferred via the AM fungal hyphae towards the roots, standardized per unit of root biomass, was highest in the QS73 treatment, significantly (*p* < 0.05) higher than for the L1/4, MA2, and STSI isolates (Fig. 3C). The amount of ^15^N detected in the hyphae (standardized per unit hyphal biomass) was highest for the QS81 genotype, followed by STSI and QS73 (Fig. 3D). Across all the fungal treatments, a negative correlation between bacterial and *Rhizophagus* abundances was detected in the BAC-BOX (Fig. S1). Additionally, bacteria were less abundant upon presence of protists (Table S2).

### Experiment 2

**Fig. 4:**
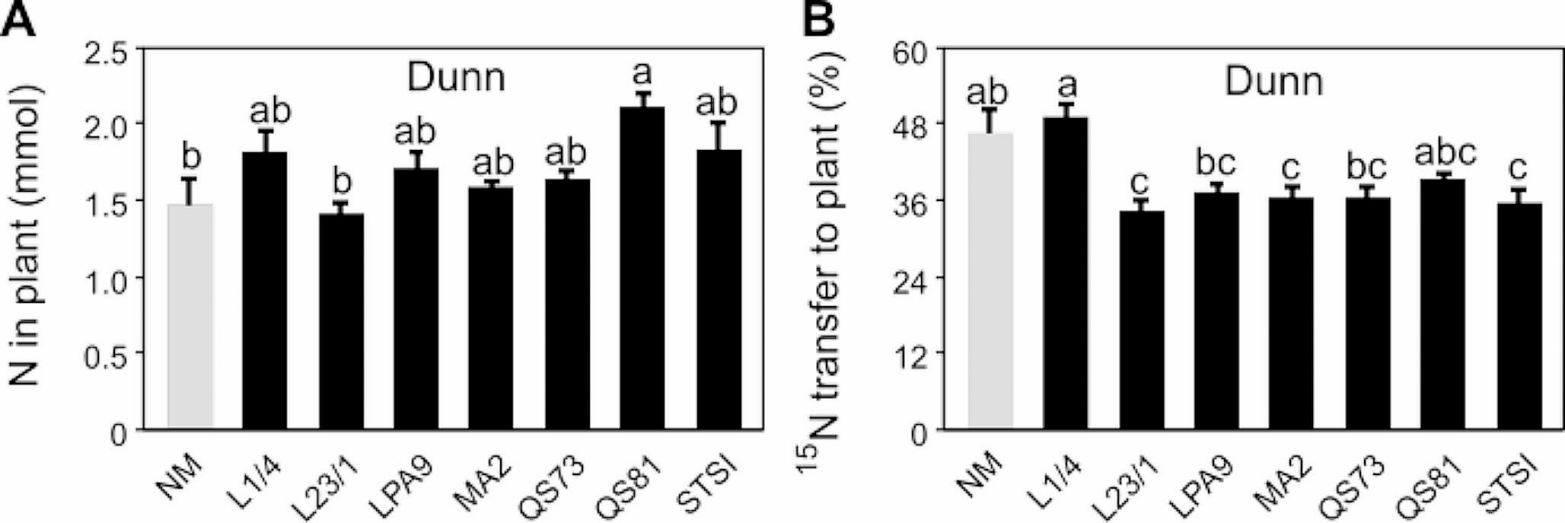
Total nitrogen (N) content in plant biomass (**A**), and the quantity of ^15^N transferred from the ^15^N-labeled patch to the plant (**B**) in **Exp 2**. Treatments include non-mycorrhizal (NM) control and seven genotypes of *Rhizophagus irregularis* (L1/4, L23/1, LPA9, MA2, QS73, QS81, and STSI). Bars represent means (*n* = 4), error bars represent standard errors. Bars topped by the same letter do not differ significantly according to the non-parametric Kruskal-Wallis test (followed by a Dunn’s pairwise multiple comparisons procedure to separate treatment medians, *p* < 0.05)

**Fig. 5:**
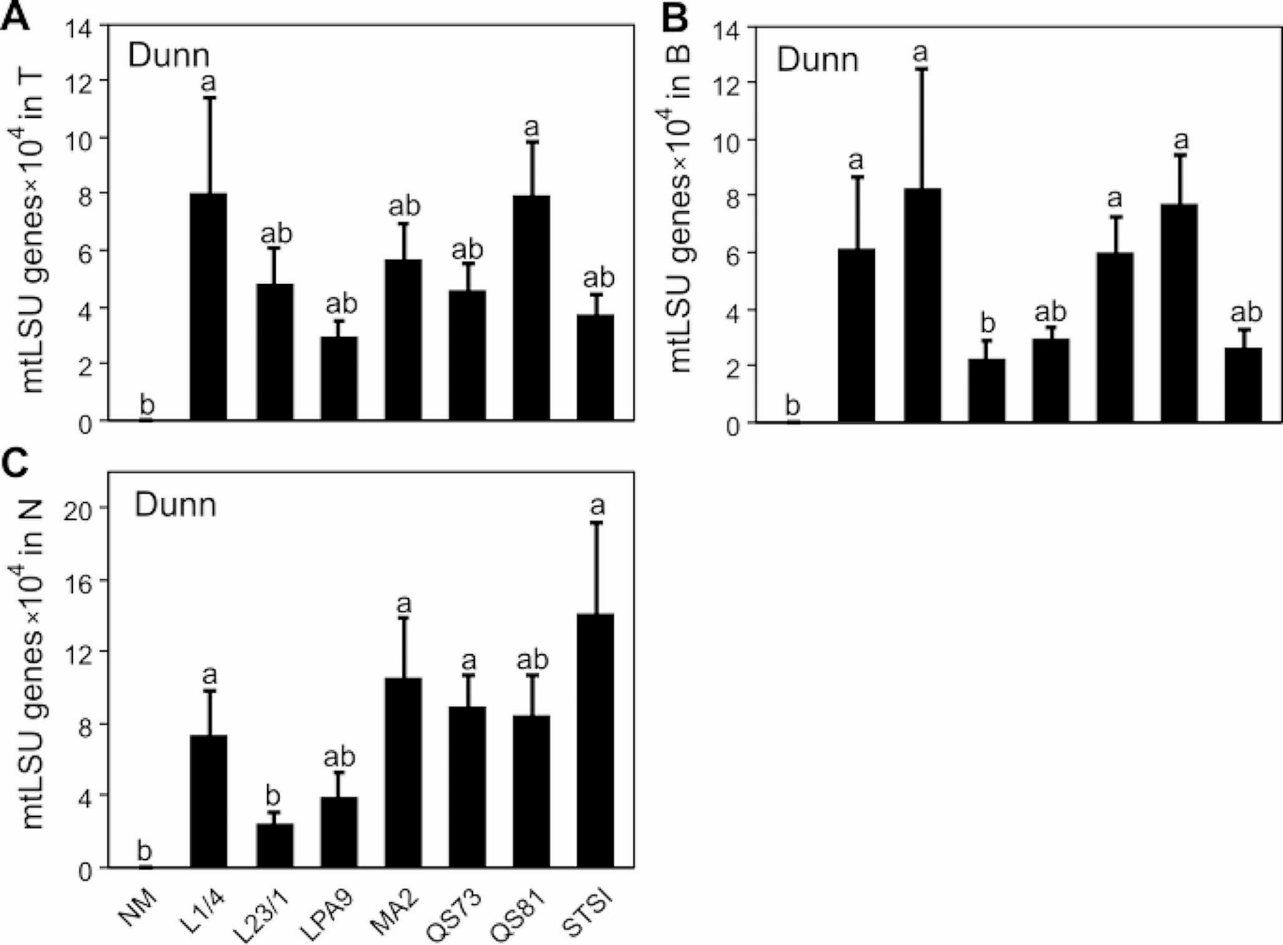
*Rhizophagus irregularis* abundance in rhizosphere (**A**), buffer zone (**B**), and the ^15^N-labeling zone (**C**) of **Exp 2**, as per quantitative real-time PCR with the mt5 marker (mitochondrial large subunit, mtLSU, gene copies per mg substrate). Treatments include non-mycorrhizal (NM) control and seven genotypes of *Rhizophagus irregularis* (L1/4, L23/1, LPA9, MA2, QS73, QS81, and STSI). Bars represent means (*n* = 4), error bars represent standard errors. Bars topped by the same letter do not differ significantly according to the non-parametric Kruskal-Wallis test (followed by a Dunn’s pairwise multiple comparisons procedure to separate treatment medians, *p* < 0.05). Values for the NM treatment were all set at zero because no or spurious values were detected

There was no significant effect of the AM fungal inoculation on root, shoot or plant biomass (Figs. S2A-B), but all the AM fungal genotypes significantly increased P uptake of the plants (Fig. S2C-D). Only the QS81 genotype increased N accumulation in plants as compared to the NM treatment (Fig. 4A). Interestingly, this was not because of improved transfer of N to shoots (Fig. S3A), but rather because of elevated N accumulation in the roots (Fig. S3B). AM fungal inoculation did not increase ^15^N transfer from the hyphosphere zone to the plants (Fig. 4D). In fact, the NM treatment had the highest ^15^N content in the shoots among all the fungal treatments (Fig. S3C), whereas three out of seven AM fungal genotypes had higher ^15^N content in the roots than the NM treatment, namely the L1/4, MA2, and QS81 genotypes (Fig. S2D). With respect to the total ^15^N balance, the residual amount of ^15^N detected in the hyphosphere compartment was not significantly different among the fungal treatments (Fig. 5A), the (calculated) ^15^N loss from the pots was the highest in L23/1 and lowest in L1/4 genotype treatments, whereas the NM treatment was not different from either of the latter two AM fungal treatments (Fig. S5B).

### Root colonization in Exp2

The fractional root lengths colonized by AM fungal hyphae and arbuscules were not significantly different among the different AM fungal genotype treatments (Fig. S4A, B), whereas root occupancy by vesicles was significantly lower in QS81 than in L1/4, L23/1, and MA2 treatments (Fig. S4C).

Gene copy numbers of *Rhizophagus* mtLSU per unit substrate weight in the rhizosphere zone were not significantly different among AM fungal treatments (Fig. 5A). In the buffer zone, LPA9 showed the lowest mtLSU gene copy numbers, significantly lower than four other AM fungal genotypes (Fig. 5B). In the hyphosphere zone, L23/1 genotype had the lowest mtLSU gene copy numbers, significantly lower than four other genotypes (Fig. 5C).

### Hyphal exploration index in experiments 1 and 2

**Fig. 6:**
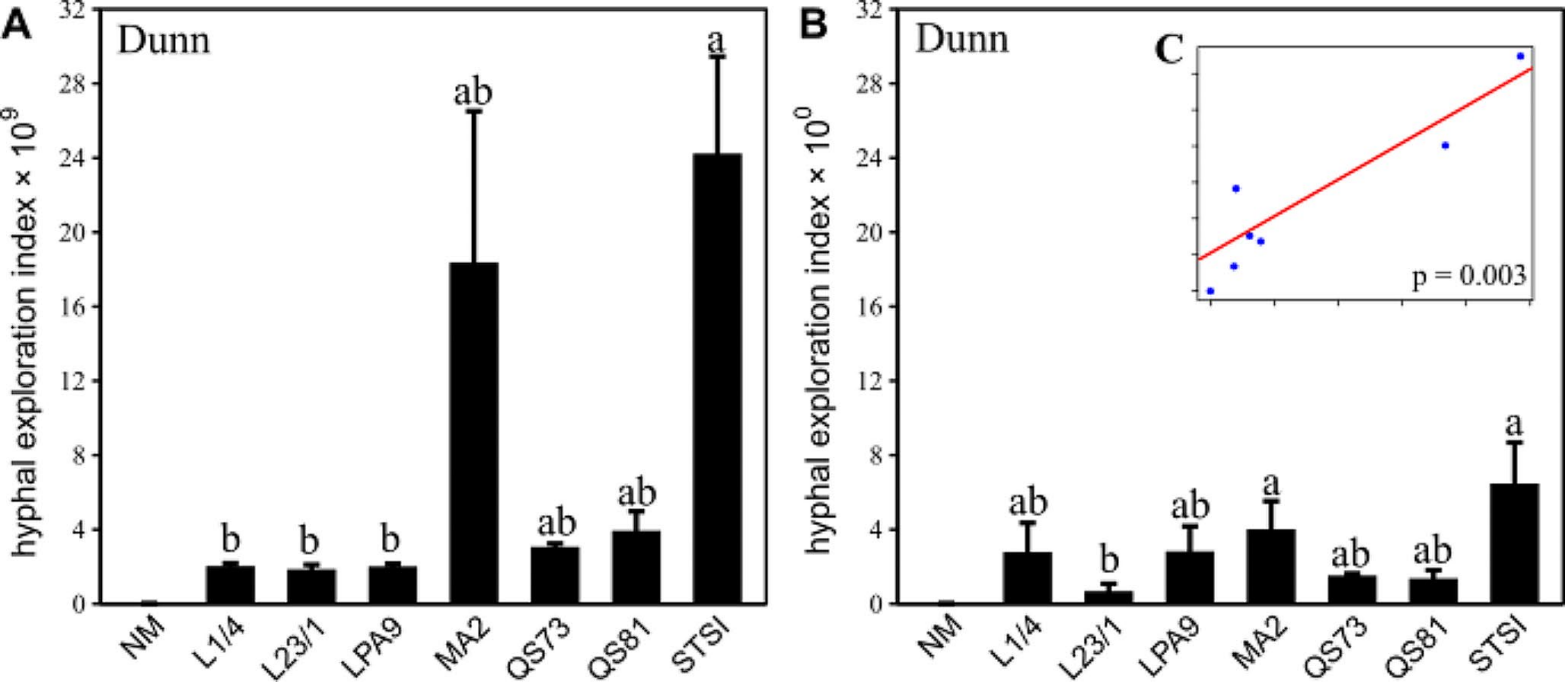
Hyphal exploration index (i.e., abundance of *Rhizophagus* in the BAC-BOX as per qPCR/ abundance in the root-free zone as per mycelium dry weight in **Exp 1** (**A**). Hyphal exploration index in **Exp 2** (**B**) was calculated as the ratio of *Rhizophagus* abundance (measured by qPCR) in the labeling zone divided by *Rhizophagus* abundance (also measured by qPCR) in the buffer zone. The insert (**C**) illustrates correlation of mean values of hyphal exploration index for the individual fungal genotype treatments measured in **Exp 1** and **Exp 2** (exclusing the non-mycorrhizal (NM) control). Treatments in panels A and B include NM control and seven genotypes of *Rhizophagus irregularis* (L1/4, L23/1, LPA9, MA2, QS73, QS81, and STSI). Bars represent means (12 to 14 biological replicates in Exp 1 and 4 replicates in Exp 2), error bars represent standard errors. Bars topped by the same letter do not differ significantly according to the non-parametric Kruskal-Wallis test (followed by a Dunn’s pairwise multiple comparisons procedure to separate treatment medians, *p* < 0.05). Values for NM treatment were all set at zero because no or spurious values were detected, and the NM treatment was exluded from the statistical comparisons

The HEI in Exp 1 was highest for the STSI genotype, followed by MA2, QS73, and QS81 genotypes, and the values remained low for the L1/4, L23/1 and LPA9 genotypes (Fig. 6A). In the Exp 2, the HEI was the highest for the STSI genotype, which showed similar values to MA2, with both showing higher HEI than the L23/1 genotype (Fig. 6B). A significant correlation (*p* = 0.003) was observed between the mean HEI values per treatment in Exp 1 and Exp 2 (Fig. 6C).

### Microbial communities in experiment 2

While community compositions of the three microbial guilds (bacteria, fungi, and protists) were well differentiated by compartments (i.e., comparing the rhizosphere and the hyphosphere zones, Fig. S6, the different AM fungal genotypes did not differentially affect the microbial communities (Fig. S6, Tables S2 and S3).

## Discussion

Previous studies revealed that intraspecific genetic variability within AM fungi can influence the performance of the host plant (Koch et al. 2017). Chaudhary et al. (2022) defined those variable mycorrhizal traits as being morphological, physiological, or phenological between plants and mycorrhizal fungus associates. In our study, we specifically focused on examination of hyphal explorative traits (the physiological/functional traits), specifically represented by the hyphal exploration index (HEI), by employing a range of *Rhizophagus* genotypes. We particularly looked for systematic functional differences among the genotypes, detectable both in a model in vitro system and in a pot experiment. Further, we aimed to link these traits to their effects on externally supplied organic N utilization by the fungus and the associated plant, in concert with other microorganisms such as bacteria and protists. This goes a step further than Koch et al. (2004), who observed differences in hyphal length and spore formation among 16 single-spore isolates of *Rhizophagus*. Our results provide additional evidence to establish potential relationships between HEI and utilization/transportation of N from a root-free zone towards the root, and also in the roles which the soil microbiome plays in these processes.

As anticipated, mycelium from all the AM fungal genotypes included in this study transferred significant amounts of ^15^N from the BAC-BOX to roots compared to the NM control in Exp 1. In Exp 2, mycorrhizal inoculation consistently exhibited a positive impact on plant P acquisition, but its effect on plant N acquisition primarily was manifested within the roots (Fig. S3). The differing abilities of different AM fungal genotypes (such as QS81 vs. L23/1, Fig. S3) to assist in plant N acquisition were consistent with the findings of Exp 1 (see biomass data in Fig. 3D and N data following the same pattern, analyses not shown, data supplied as a supplement), strongly indicating that the extent of N exploration was dependent on the identity of the AM fungal genotypes rather than the exact experimental set-up.

Consistent with the above, there were notable differences in the development of the hyphae among the AM fungal genotypes. For instance, LPA9 systematically produced a greater mycelium biomass in the in vitro system (per microcosm) as compared with the other AM fungal genotypes. Noticeably, despite its higher mycelium biomass, LPA9 did not acquire a correspondingly higher percentage of ^15^N from the organic N source compared to QS81 and other genotypes with inherently lower mycelium biomass production (Fig. 3). Further, although not supported statistically, LPA9 tended to cause somewhat lower N acquisition of its plant host than QS81 in Exp 2 (Fig. 4), although this could have also been caused by different developmental patterns of the different AM fungal genotypes in the root-free zones of Exp 1 and Exp 2 (please compare Figs. 3A and 5B for more details).

This apparent disconnect between AM fungal biomass and nutrient acquisition and transport towards roots from the root-free zone, whether retained within the mycelium or transferred to the roots, may be explained by certain AM-plant relationships exhibiting “cheating” tendencies (Jones et al. 2015) with the fungi hoarding a significant proportion of resources in hyphae and potentially competing with their host plants for those resources (Kiers et al. 2011; Püschel et al. 2016; Kokkoris and Hart 2019). Additionally, different AM fungi might have their nutrient transporters expressed differently and with different nutrient affinities. Currently there is too little information regarding what are the limiting factors of nutrient acquisition per unit hyphal length of different AM fungi to gain deeper mechanistic understanding of the underlying factors of the observed patterns.

Nevertheless, it appears that such nutrient competition between the AM fungi and the host plant as outlined above was not primarily triggered by environmental factors. This is because both experiments yielded similar HEI (such as high values recorded for STSI and MA2 genotypes), and the experiments (at least Exp 1) were not particularly limited in nutrient availability. Interestingly, preliminary analysis of genetic differences among the different isolates (e.g., Fig. S7) and/or grouping the isolates based on different criteria (Table S3, Table S4) did not provide an unequivocal answer as to what underlying features of the different AM fungal genotypes caused the differences in AM hyphal nutrient exploration or associated microbial community composition.

Previous studies have uncovered significant genetic diversity among genotypes of *R. irregularis* (Jansa et al. 2002; Mathieu et al. 2018) and also have shed light on nuclear organization within this important AM fungus species (Corradi et al. 2007; Chen et al. 2018; Yildirir et al. 2022). Two distinct genetic categories within *R. irregularis* strains namely the *dikaryons* and *homokaryons* have been reported (Ropars et al. 2016; Sperschneider et al. 2023). Dikaryotic strains have been observed to exhibit characteristics such as rapid extraradical hyphal growth and the ability to establish intricate extraradical hyphal networks. In contrast, homokaryotic strains tend to show a higher rate of spore germination than dikaryotic strains (Serghi et al. 2021). The seven *R. irregularis* genotypes included in the experimentation reported here all were homokaryons (determined by PCR and Sanger sequencing), thus genotypes within homokaryons also could have different hyphal nutrient exploration traits.

In the process of organic N utilization within the root-free zone of Exp 1, a noteworthy negative correlation between AM fungal abundance and bacterial abundance was observed (Fig. S1). It is worth noting that an equal quantity of bacterium (*Paenibacillus*) was introduced into each BAC-BOX upon bacterial inoculation. Thus, such a negative correlation between fungal and bacterial abundances suggests that competition for N was primarily driven by the AM fungi, in agreement with previous research (Bukovská et al. 2018). In other words, an environment in which AM fungi have depleted the available N resources tends to reduce bacterial development. Notably, in Exp 2, where a diverse microbial community was present (in contrast to Exp 1 which only was inoculated with a single bacterium, *Paenibacillus*), we did not detect significant shifts in the microbial communities, including bacteria, fungi, or protists, in response to the different AM fungal genotypes (Table S3, Table S4), although the ^15^N utilization was generally higher in Exp 2 than in Exp 1.

In summary, our findings highlight that the HEI differed among different genotypes belonging to *Rhizophagus irregularis*, and in particular those genotypes did exhibit distinct strategies for soil N exploitation, consistently in both in vitro and pot experiments.

## Electronic supplementary material

Below is the link to the electronic supplementary material.

Supplementary Material 1

Supplementary Material 2

Supplementary Material 3

Supplementary Material 4

## Data Availability

The sequences were deposited in the Sequence Read Archive of the NCBI under the accession number PRJNA977454.
