# Correction: Unraveling the diversity of hyphal explorative traits among *Rhizophagus irregularis* genotypes

**DOI:** 10.1007/s00572-024-01173-5

**Published:** 2024-11-13

**Authors:** Daquan Sun, Martin Rozmoš, Vasilis Kokkoris, Michala Kotianová, Hana Hršelová, Petra Bukovská, Maede Faghihinia, Jan Jansa

**Affiliations:** 1 Institute of Microbiology, Czech Academy of Sciences, Vídeňská, 14220, Praha 4, 1083 Czech Republic; 2 Vrije Universiteit Amsterdam, Amsterdam Institute for Life and Environment (A-LIFE), De Boelelaan 1108, Amsterdam, NL-1081HZ The Netherlands; 3 Department of Plant Pathology, Entomology, and Microbiology, Iowa State University, 2213 Pammel Dr, Ames, IA 50011 USA

**Correction to: Mycorrhiza (2024) 34:303–316**

10.1007/s00572-024-01154-8

In the published original article, some errors.

These errors can be found on page 4, 5, 7–11.

An updated supplementary file has been provided as well.

The original article has been corrected.

