# Supplementary material for: Unraveling the diversity of hyphal explorative traits among Rhizophagus irregularis genotypes
Source: Mycorrhiza. 2024 Jun 3;34(4):303–16. doi: 10.1007/s00572-024-01154-8 (PMC11283409; doi:10.1007/s00572-024-01154-8)
Supplement: Supplementary file 2 — Supplementary Material 2 [file 572_2024_1154_MOESM2_ESM.pdf]

Unraveling the diversity of hyphal explorative traits among *Rhizophagus irregularis* genotypes

Daquan Sun[a]*, Martin Rozmoš[a], Vasilis Kokkoris[b], Michala Kotianová[a], Hana Hršelová[a], Petra Bukovská[a], Maede Faghihinia[a,c], Jan Jansa[a]

[a] Institute of Microbiology, Czech Academy of Sciences, Vídeňská 1083, 14220 Praha 4, Czech Republic

[b] Vrije Universiteit Amsterdam, Amsterdam Institute for Life and Environment (A-LIFE), De Boelelaan 1108, NL-1081HZ Amsterdam, The Netherlands

[c] present address: Department of Plant Pathology, Entomology, and Microbiology, Iowa State University, 2213 Pammel Dr, 50011 Ames, IA, United States

Corresponding author: *daquan.sun@biomed.cas.cz; daquansun1010@gmail.com

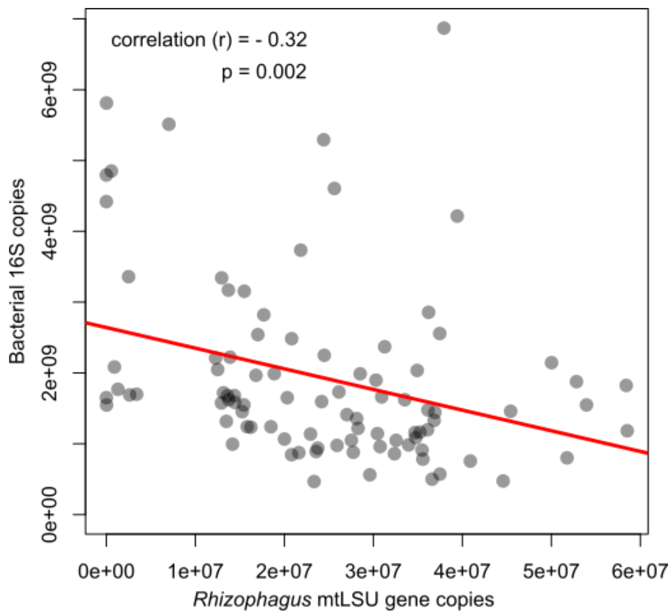

Fig. S1 Correlation between bacterial 16S rRNA gene copy numbers per microcosm and *Rhizophagus irregularis* mtLSU gene copies (mt5 marker) per microcosm in the BAC-BOX of **Exp 1**.

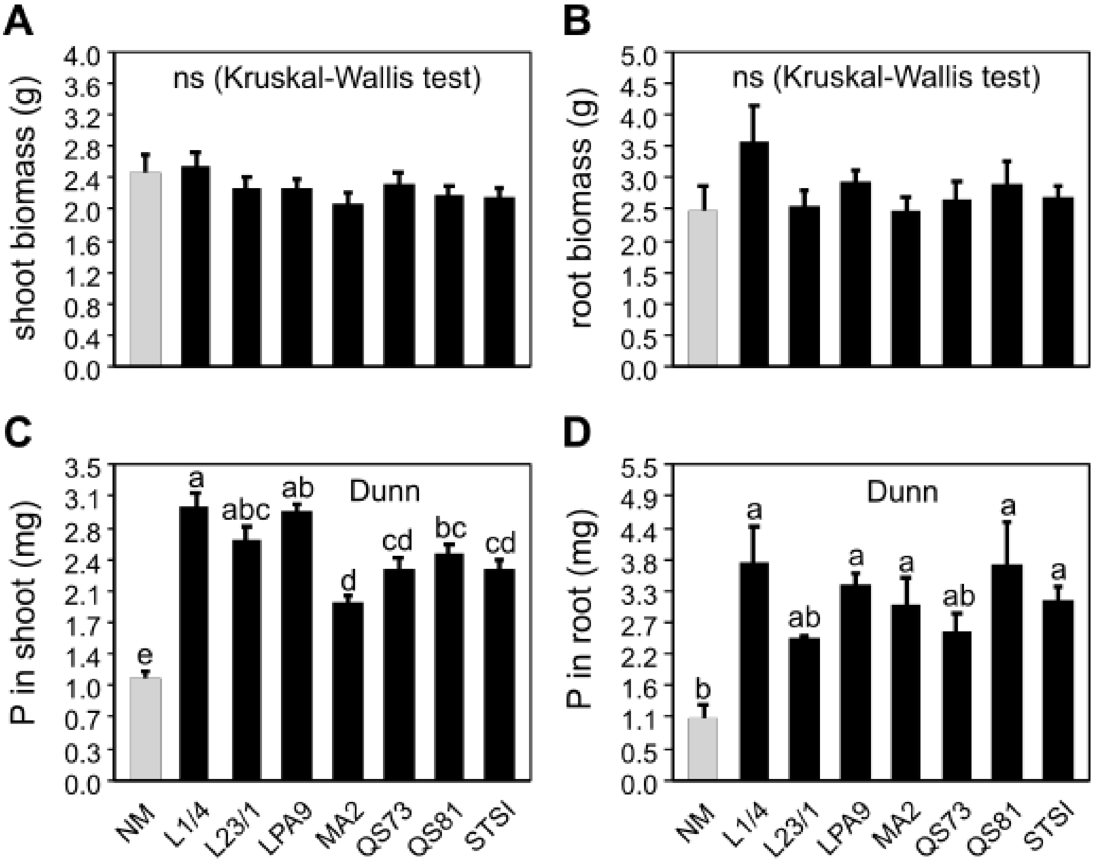

Fig. S2 Plant biomass in shoots (A) and roots (B) per pot, and phosphorus (P) content in shoots (C) and roots (D) in **Exp 2**. Treatments include non-mycorrhizal (NM) control and seven genotypes of *Rhizophagus irregularis* (L1/4, L23/1, LPA9, MA2, QS73, QS81, and STSI). Bars represent means (n = 4), error bars represent standard errors. Bars topped by the same letter do not differ significantly according to the non-parametric Kruskal-Wallis test (followed by a Dunn´s pairwise multiple comparisons procedure to separate treatment medians, $p < 0.05$). ns indicates no statistical differences among the treatments.

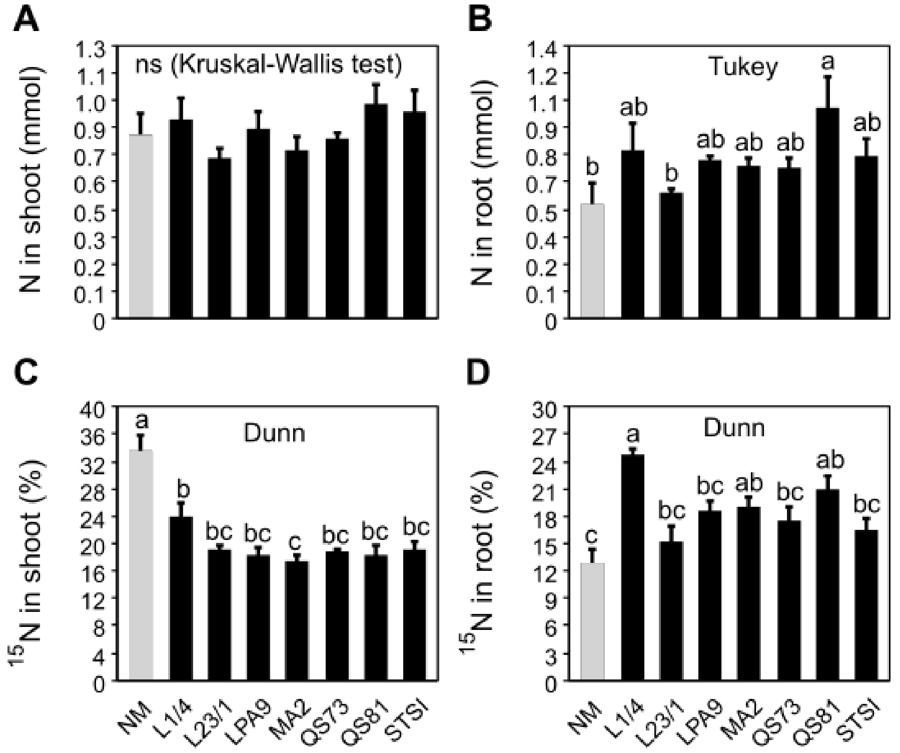

Fig. S3 Nitrogen (N) content in shoots (A) and roots (B) per pot, and the quantity of ^{15}N transferred from the labeling compartment to shoots (C) and roots (D) in **Exp 2**. Treatments include non-mycorrhizal (NM) control and seven genotypes of *Rhizophagus irregularis* (L1/4, L23/1, LPA9, MA2, QS73, QS81, and STSI). Bars represent means (n = 4), error bars represent standard errors. Bars topped by the same letter do not differ significantly according to the non-parametric Kruskal-Wallis test (followed by a Dunn´s pairwise multiple comparisons procedure to separate treatment medians, $p < 0.05$) or as per one-way ANOVA (followed by a Tukey-HSD test, $p < 0.05$). ns indicates no statistical differences among the treatments.

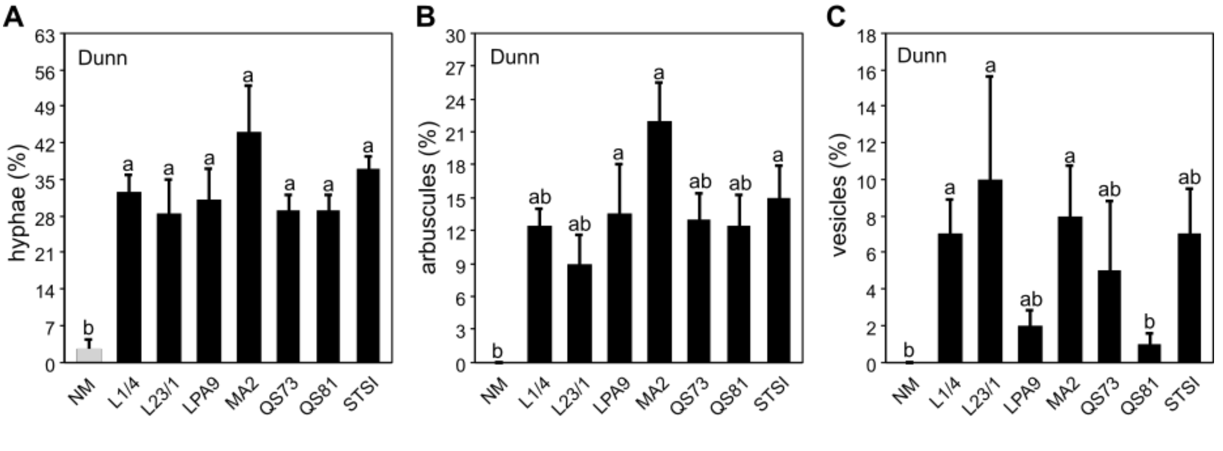

Fig. S4 Colonization by arbuscular mycorrhizal (AM) fungal structures of roots in **Exp 2**. Fractional root length colonization by AM fungal hyphae (A), arbuscules (B), and vesicles (C) is shown. Treatments include non-mycorrhizal (NM) control and seven genotypes of *Rhizophagus irregularis* (L1/4, L23/1, LPA9, MA2, QS73, QS81, and STSI). Bars represent means ($n = 4$), error bars represent standard errors. Bars topped by the same letter do not differ significantly according to the non-parametric Kruskal-Wallis test (followed by a Dunn's pairwise multiple comparisons procedure to separate treatment medians, $p < 0.05$).

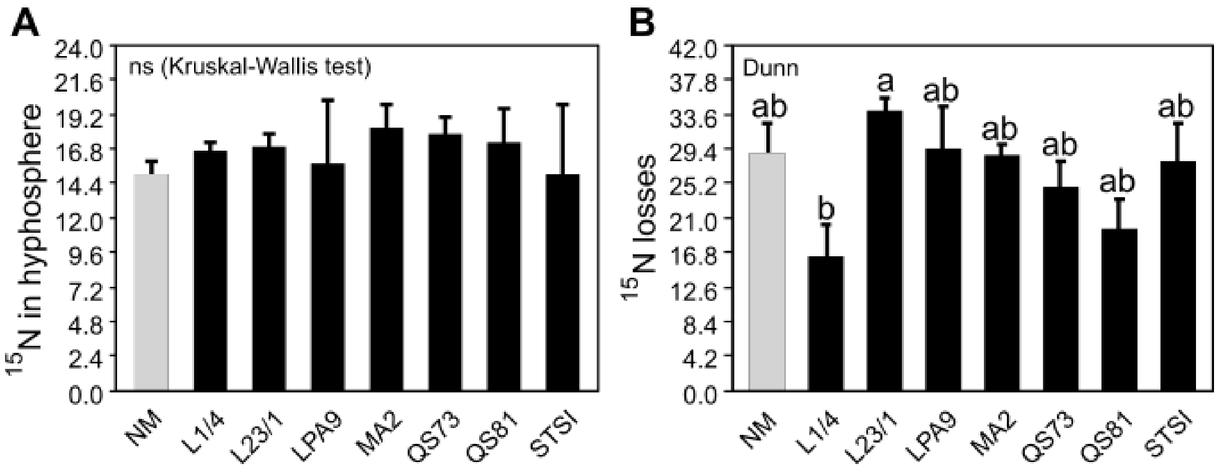

Fig. S5 Measured residual ^{15}N in the labeling compartment (A) and calculated ^{15}N loss (B) from the pots in **Exp 2**. Percentage values referring to the total amount of ^{15}N isotope supplied with the labeled chitin are shown. Treatments include non-mycorrhizal (NM) control and seven genotypes of *Rhizophagus irregularis* (L1/4, L23/1, LPA9, MA2, QS73, QS81, and STSI). Bars represent means (n = 4), error bars represent standard errors. Bars topped by the same letter do not differ significantly according to the non-parametric Kruskal-Wallis test (followed by a Dunn's pairwise multiple comparisons procedure to separate treatment medians, $p < 0.05$). ns indicates no significant differences among the treatments.

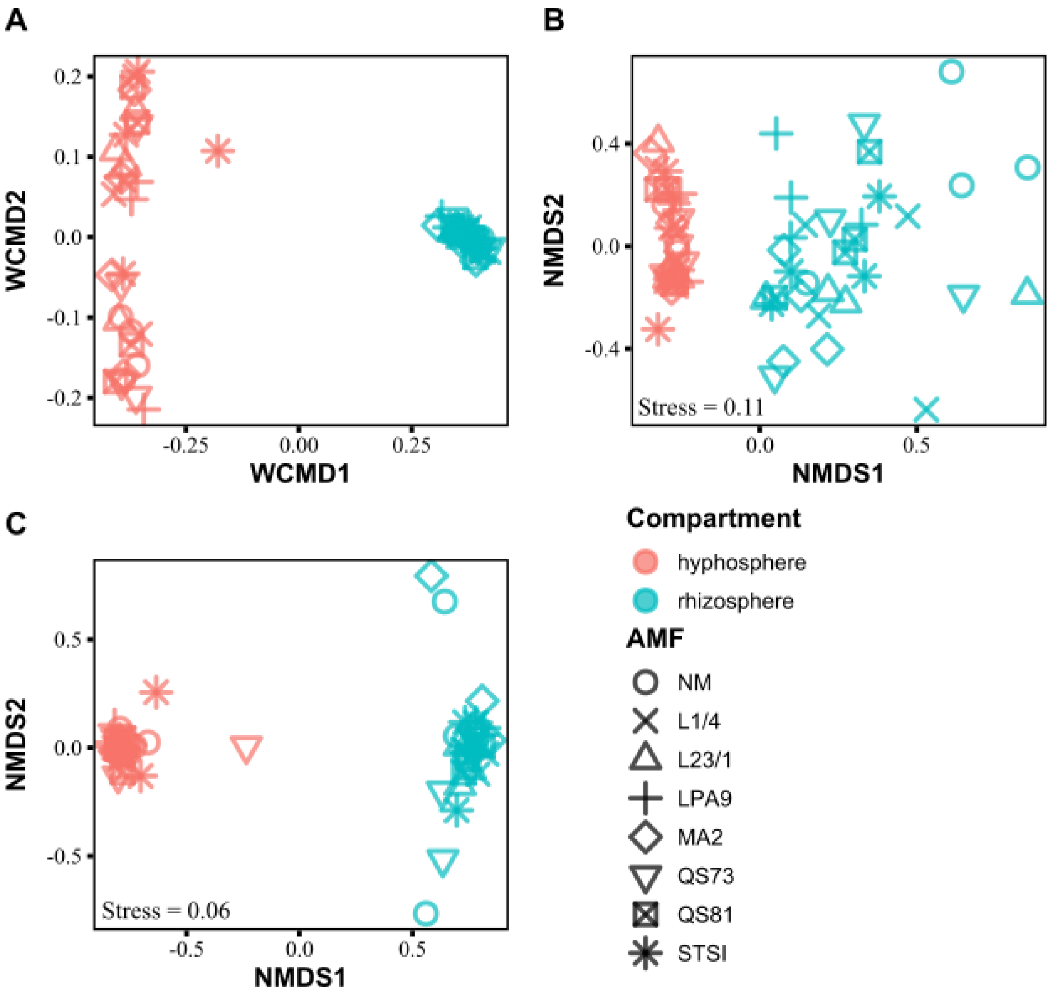

Fig. S6 Weighted Classical (Metric) Multidimensional Scaling (WCMD) and Nonmetric multidimensional scaling (NMDS) ordinations of Bray-Curtis dissimilarities of microbial communities in the ^{15}N labeling compartment (hyphosphere or N compartment, according to Fig. 1) and rhizosphere in **Exp 2**. A: bacterial community; B: fungal community; and C: protistan community. The treatments include non-mycorrhizal (NM) control and seven genotypes of *Rhizophagus irregularis* (L1/4, L23/1, LPA9, MA2, QS73, QS81, and STSI).

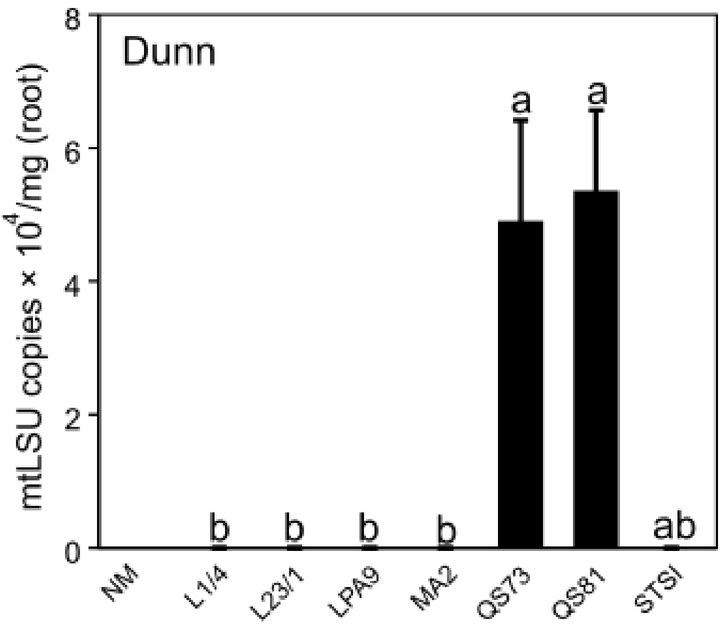

Fig. S7 Mitochondrial large ribosomal subunit gene copies of *Rhizophagus* type QS81, quantified by a newly designed QS81 marker in roots of plants in **Exp 2**, considering non-mycorrhizal (NM) control and seven genotypes of *Rhizophagus irregularis* (L1/4, L23/1, LPA9, MA2, QS73, QS81, and STSI). Bars represent means ($n = 4$), error bars represent standard errors. Bars topped by the same letter do not differ significantly according to the non-parametric Kruskal-Wallis test (followed by a Dunn´s pairwise multiple comparisons procedure to separate treatment medians, $p < 0.05$). NM treatment was excluded from the statistical analysis because only one replicate sample was analyzed.
