# Supplementary material for: Unraveling the diversity of hyphal explorative traits among Rhizophagus irregularis genotypes
Source: Mycorrhiza. 2024 Jun 3;34(4):303–16. doi: 10.1007/s00572-024-01154-8 (PMC11283409; doi:10.1007/s00572-024-01154-8)
Supplement: Supplementary file 4 — Supplementary Material 4 [file 572_2024_1154_MOESM4_ESM.pdf]

**Unraveling the diversity of hyphal explorative traits among *Rhizophagus irregularis* genotypes**

Daquan Sun[a]*, Martin Rozmoš[a], Vasilis Kokkoris[b], Michala Kotianová[a], Hana Hršelová[a], Petra Bukovská[a], Maede Faghihinia[a,c], Jan Jansa[a]

[a] Institute of Microbiology, Czech Academy of Sciences, Vídeňská 1083, 14220 Praha 4, Czech Republic

[b] Vrije Universiteit Amsterdam, Amsterdam Institute for Life and Environment (A-LIFE), De Boelelaan 1108, NL-1081HZ Amsterdam, The Netherlands

[c] present address: Department of Plant Pathology, Entomology, and Microbiology, Iowa State University, 2213 Pammel Dr, 50011 Ames, IA, United States

Corresponding author: *daquan.sun@biomed.cas.cz; daquansun1010@gmail.com

**Table S1** List of arbuscular mycorrhizal fungal genotypes used in this study, their origin and year of isolation.

| Genotype ID | Provenance | Provider | origin | isolated in | previous coding |
|---|---|---|---|---|---|
| LPA9 | BEG[#] Dijon via SymbioM | Martin Rozmoš | Greece | 1980 | BEG236 |
| L1/4 | Jansa lab, single spore isolate | Martin Rozmoš | Czech Republic | 2015 | |
| L23/1 | Jansa lab, single spore isolate | Martin Rozmoš | Czech Republic | 2015 | |
| MA2 | INOQ[§] | Louis Mercy | Mayotte | 2008 | |
| QS73 | INOQ | Louis Mercy | Germany* | 2008 | |
| QS81 | INOQ | Louis Mercy | Germany* | 2008 | |
| STSI | INOQ | Louis Mercy | Germany** | 2018 | |

[#] The International Bank for the Glomeromycota (www.i-beg.eu/)

[§] INOQ GmbH, Solkau 2, 29465 Schnega, Germany (inoq.de/en/)

*Originally isolated by Hermann Bothe from zinc violet from a field site near Breinigerberg, Germany sometimes between 2002 and 2005. These (sister) strains were transferred into monoxenic cultures in the year 2008.

**Culture established from field potato roots collected at Loitze (Germany) – sandy soil, poor in nitrogen and phosphate – in the year 2018.

**Table S2** Two-way ANOVA testing the effect of different genotypes of *Rhizophagus* (AM) and inoculation with a protist into the BAC-BOX (protist, yes or no), and their interaction (AM × protist) on several variables measured in **Exp 1**. Data were ranked before two-way ANOVA for tackling non-normality issues. F and p values are indicated. Significant p values are shown in bold.

| | AM (df = 7) | Protist (df = 1) | AM × protist (df = 7) |
|---|---|---|---|
| root DW | 9.537 (< **0.001**) | 4.655 (**0.034**) | 1.752 (0.107) |
| mycelium DW | 124.617 (< **0.001**) | 3.106 (0.081) | 1.442 (0.198) |
| $^{15}N$ transfer to roots (%) | 11.205 (< **0.001**) | 0.297 (0.587) | 0.492 (0.838) |
| $^{15}N$ transfer to hyphae (%) | 113.046 (< **0.001**) | 0.447 (0.506) | 2.726 (**0.013**) |
| $^{15}N$ transfer to roots per root biomass | 28.428 (< **0.001**) | 0.014 (0.906) | 0.648 (0.715) |
| $^{15}N$ transfer to hyphae per hyphae biomass | 63.638 (< **0.001**) | 1.434 (0.234) | 4.986 (< **0.001**) |
| *Rhizophagus* mtLSU gene copies per BAC-BOX | 17.188 (< **0.001**) | 0.123 (0.727) | 1.387 (0.220) |
| bacterial 16S gene per BAC-BOX | 10.825 (< **0.001**) | 41.599 (< **0.001**) | 2.005 (0.063) |
| protistan 18S gene per BAC-BOX | 2.602 (**0.017**) | 368.967 (< **0.001**) | 2.380 (**0.028**) |
| hyphal exploration index | 15.831 (< **0.001**) | 0.020 (0.888) | 1.617 (0.141) |

DW – dry weight, mtLSU – mitochondrial large ribosomal subunit

**Table S3** Permutational analysis comparing the effects of pot compartment including rhizosphere and the hyphosphere (N) compartments in **Exp 2**, and all inoculation treatments and the interaction of pot compartment and mycorrhizal inoculation (com*AM) on the beta diversity (Adonis, Bray) of bacterial, fungal and protistan communities. $R^2$ and p-values are indicated. Significant p values are shown in bold.

| | compartment | AM | com * AM |
|---|---|---|---|
| Bacteria | 0.768 (< **0.001**) | 0.036 (0.152) | 0.034 (0.163) |
| Fungi | 0.482 (< **0.001**) | 0.066 (0.312) | 0.057 (0.464) |
| Protists | 0.741 (< **0.001**) | 0.040 (0.148) | 0.036 (0.203) |

**Table S4** Permutational analysis testing the effects of different genotype groups of *Rhizophagus* (group 1 and group 2) on beta diversity (Adonis, Bray) of bacterial, fungal and protistan communities in the rhizosphere and the hyphosphere (N) compartments in **Exp 2**. Group 1 includes data structured with treatments clustered in 3 categories: NM, responsive AM with respect to HEI in both experiments (MA2, STSI), or non-responsive AM genotypes (LPA9, L1/4, L23/1, QS73, QS81); Group 2 includes data structured with treatments clustered in 4 categories NM, responsive AM (MA2, STSI), less -responsive AM (LPA9, L1/4), or others (L23/1, QS73, QS81). $R^2$ and p-values are indicated.

| | Group 1 | Group 2 |
|---|---|---|
| Bacteria | 0.107 (0.066) | 0.175 (0.103) |
| Fungi | 0.014 (0.942) | 0.047 (0.949) |
| Protists | 0.075 (0.322) | 0.164 (0.144) |
